# House feeding system improves the estrus rate in yaks (*Bos grunniens*) by increasing specific fecal microbiota and myo-inositol content in serum

**DOI:** 10.3389/fmicb.2022.974765

**Published:** 2022-09-07

**Authors:** Yanbin Zhu, Xin Li, Lousang zhaxi, Suolang zhaxi, Suolang, Ciyang, Guangming Sun, Cidan yangji, Basang wangdui

**Affiliations:** ^1^Linzhou Animal Husbandry and Veterinary Station, Lhasa, China; ^2^Institute of Animal Science and Veterinary, Tibet Academy of Agricultural and Animal Husbandry Sciences, Lhasa, China

**Keywords:** yaks, house feeding, grazing, the estrus rate, fecal microbiota, serum metabolomics

## Abstract

Grazing (G) yaks (*Bos grunniens*) are generally of low fertility, which severely limits the income of local pastoralists. However, we recently found that yaks had a 52% higher estrus rate in house feeding (HF) than in G. Gas chromatography-mass spectrometry (GC-MS) and 16S rRNA gene sequencing were used to analyze serum metabolites and fecal microbiota of 20 rutting yaks in the G and HF systems, respectively, to explain this phenomenon. The results showed that 73 total metabolites differed significantly (*p* < 0.05 and VIP > 1) between the G and HF systems. In the HF system, 53 were upregulated and 20 were downregulated compared with the G system. Organic oxygen compounds, organic acids and their derivatives, and lipids and lipid-like molecules were the most common differential metabolites. The Kyoto Encyclopedia of Genes and Genomes (KEGG) pathway mapper revealed that 25 metabolic signaling pathways differed significantly between the two systems. The top three enriched pathways included central carbon metabolism in cancer, aminoacyl–tRNA biosynthesis, and ABC transporters. The 16S rRNA gene sequencing data showed no significant differences in Chao 1 index between the two systems. According to principal component analysis (PCA), the HF and G systems were distinctly and separately clustered in terms of fecal microbiota distribution. The G system showed significantly higher abundances of *Firmicutes*. The HF system showed significantly higher abundances of *Alistipes, Treponema*, and *Rikenellaceae_ RC9_ gut_ group*. Pearson's correlation analysis and core network analysis revealed that *Rikenellaceae_RC9_ gut_ group, Alistipes*, and *Treponema* were positively correlated with myo-inositol and formed the core bacteria. In summary, the HF system promoted the estrus rate and changed the composition of yak fecal microbiota and serum metabolites. Increased estrus rate might be obtained due to enhanced myo-inositol content in yak serum *via* the HF system. Correlation analysis suggested that myo-inositol content might also be partly increased *via* yak-specific fecal microbiota, contributing to the estrus rate. These findings could lead to a novel therapeutic strategy for G yaks due to their low estrus rate.

## Introduction

Yaks (*Bos grunniens*) are major indigenous ruminants distributed mainly in the Qinghai-Tibet plateau in high-altitude areas between 2,500 and 6,000 m (Qiu et al., 2012; Xiong et al., 2015). Through long evolution, yaks have adapted to live in high altitude and low oxygen environments. They thrive and reproduce in thin air, cold temperatures, and short grass (Lan et al., 2016; Ma et al., 2020). Tibetan nomads rear them primarily for milk, meat, wool, fuel, and other necessities (Fu et al., 2014a; Guo et al., 2014). Therefore, it is also an important source of income for local pastoralists (Guo et al., 2014).

However, yaks commonly face the problem of low fertility. Data suggest that the average reproduction rate of yaks is only 48.61%, which represents one birth in 2 years or two births in 3 years. Moreover, more than 90% of postpartum yaks cannot rut during the estrus season (Fu et al., 2014b; Xiao et al., 2014). Calving rates have remained durably low, which greatly limits herd expansion. As a result, the economic income of Tibetan herders is severely restricted. Some studies show that estrus activity is directly linked to the level of nutrients (Butler, 2000; Walker et al., 2008). Severe malnutrition reduces reproductive hormones and performance in animals (Patterson et al., 1992). The Qinghai-Tibet plateau climate experiences sharp frost in the lengthy cold season, which lasts from October until May of the following year. Highland meadows are at an average of 3,450 m above sea level and are covered by snow for about 4 months of the year. The plateau is limited in forage, resulting in lower body weight and severe malnutrition in yaks (Long et al., 1999). Thankfully, this matter is of high priority to the local government. Yaks have begun to be raised by a formulated diet in farm cowsheds under the direction of livestock technicians. This progress has significantly improved growth performance and tremendously stimulated yak estrus. Our data showed that yaks had a 52% higher estrus rate in house feeding (HF) than in grazing (G).

The ovary plays an important role in estrus due to the secretion of several hormones. However, inactive ovaries are types of anestrus caused by temporary disturbance of ovarian function and lack of periodic follicular activity (Peter et al., 2009). Some studies have found that many factors can cause or aggravate inactive ovaries (Ahmed, 2007). Among them, nutritional levels have a far-reaching impact on reproductive performance as there is a close relationship between energy balance and ovarian activity. According to Butler et al. (2006), pulsed secretion of gonadotropin-releasing hormone from the hypothalamus and luteinizing hormone from the pituitary gland were reported to be restrained by negative energy balance, thereby reducing the responsiveness of an ovary to luteinizing hormone. Previous research mainly focused on the effects of certain substances on follicular growth or ovarian activity in dairy cows (Song et al., 2019; Zhao et al., 2020a). The literature on alterations in the percentage of yak estrus in estrus due to different feeding systems is not available. However, the measure of yaks being raised in a farm cowshed significantly stimulated yak estrus. Hence, the objective of our study is to probe whether these alterations may be due to differences in feeding management systems (nutritional levels) contributing to differential metabolites in serum, which, in turn, partly affect yak estrus. Therefore, in this study, gas chromatography-mass spectrometry (GC-MS) was used to identify differential metabolites in the serum of rutting yaks under different feeding management systems. In addition, 16S rRNA gene sequencing was used to analyze the microbiota in yak feces. In anticipation, we would locate key metabolites or key bacteria, which can affect yak estrus.

## Materials and methods

### Animal and sample collection

In this study, healthy female yaks living at more than 3,700 m above sea level and in G and HF conditions were recruited from Linzhou County, Tibet, and China. A total of 100 healthy and aged 5-year-old female yaks were selected from G and HF conditions. All yaks had finished calving in the current year. Yaks in the HF system were raised by the formulated diet on a farm (Table 1). Yaks of the G system were still in the semi-wild status raised by the natural G system. To synchronize estrus during the experiment, each yak was injected with cloprostol sodium 200 μg, gorarelin 150 μg, gorarelin 150 μg, and cloprostol sodium 200 μg on days 1, 8, 9, and 10, respectively. These drugs were purchased from Ningbo Sansheng Biological Technology Co., Ltd. (Ningbo, China). A total of 10 yaks were randomly selected under G and HF conditions on day 11. It was used to collect serum and stools. Blood samples and stools were treated according to the method of Luo et al. (2021). The estrus rate from day 8 to day 14 was statistically examined using all experimental yaks. Furthermore, yaks in estrus were artificially inseminated, and the number of calves born was also recorded in the next year.

**Table 1:** Diet composition and nutrient levels^a^.

**Ingredient**	**Diet**
**composition, %**	**House feeding**
Alfalfa hay	26.20
Oatgrass	20.00
Corn	40.00
Soybean oil	0.50
Wheat bran	3.50
Soybean meal	3.50
Cottonseed meal	3.50
Rapeseed meal	0.50
Baking soda	0.50
Sodium chloride	0.30
Calcium hydrophosphate	0.50
Premix^b^	1.00
Nutrient levels, %	
DM (%)	88.37
ME (MJ/%)	9.02
CP	14.44
NDF	27.04
ADF	15.82
Total Ca	0.94
Total P	0.47

^a^DM, dry matter; ME, metabolic energy; CP, crude protein; NDF, neutral detergent fiber; ADF, acid detergent fiber.

^b^The premix provided the following per kg of diets: VA: 18,000 IU; VD3: 4,500 IU; VE 22.5 mg; VK3: 4.5 mg; VB1: 4.32 mg; VB2: 12 mg; VB6: 4.86 mg; VB12: 0.03 mg; nicotinamide: 41.58 mg; calcium pantothenate: 33.12 mg; folic acid: 1.764 mg; biotim: 0.48 mg; Cu: 20 mg; Fe: 140 mg; Zn: 140 mg; Mn: 40 mg; I: 0.5 mg; Se: 0.3 mg.

### Observation of estrus

The identification of yak estrus was performed as described by Zi et al. (2006) and Lan et al. (2016). The estrus rate was calculated by


$$\begin{array}{l} estrus\;rate=100\times \frac{Number\;of\;yaks\;in\;estrus}{Total\;number\;of\;yaks\;synchronized} \end{array}$$

### Metabonomic profiling

#### Experimental treatment

Serum was immediately frozen in liquid nitrogen (– 196°C) for GC-MS analyses. All treatments were performed in 10 biological replicates. Stool samples were stored at – 80°C for subsequent gut microbiome analyses.

#### Sample preparation

Serum samples were slowly thawed at room temperature (25°C) and collected into 2-ml microcentrifuge tubes. Approximately 0.3 ml of methanol/chloroform 3:1 (v/v) and 20 μl of L-2-chlorophenylalanine solution (1 mg ml^−1^ in distilled water) were added to the sample as an internal standard. The solutions were vortexed for 30 s, homogenized for 4 min, sonicated for 5 min (on ice), and centrifuged for 15 min at 10,000 g and 4°C. The supernatant (0.2 ml) was then transferred to a 2-ml GC-MS glass vial. Approximately 8 μl of each sample was used as a quality control (QC) sample. These samples were dried in a vacuum concentrator at room temperature. Subsequently, 70 μl of methoxylamine hydrochloride (20 mg ml^−1^ in pyridine) was added. The resultant mixture was vortexed vigorously for 2 min and incubated for 90 min at 37°C, 80 μl of BSTFA and 1% TMCS was added, and the resulting samples were incubated for 1 h at 70°C. Approximately 10 μl of a standard mixture of fatty acid methyl esters (C8–C16: 1 mg ml^−1^; C18–C24: 0.5 mg ml^−1^, in chloroform) was added to QC samples.

#### Fecal metabolic profiling of GC-MS

After sample pretreatment was completed, fecal samples were analyzed by Shanghai OE Biotech. Co., Ltd. (Shanghai, China). GC/time-of-flight mass spectrometry (TOFMS) analysis was performed using an Agilent 7890 GC system (Agilent, Santa Clara, CA, USA) coupled with a Pegasus HT time-of-flight mass spectrometer (Leco, Saint Joseph, MI, USA). The system used a DB-5MS capillary column coated with 5% diphenyl cross-linked with 95% dimethylpolysiloxane (30 m × 250 μm inner diameter, 0.25 μm film thickness; J&W Scientific, Folsom, CA, USA). A 2 μl aliquot of the analyte was injected in the splitless mode. High purity helium (>99.999%) was used as the carrier gas, the front inlet purge flow was 3 ml min^−1^, and the gas flow rate through the column was 1 ml min^−1^. The GC oven temperature was maintained at 50°C for 1 min, then increased to 305°C at a rate of 12°C min^−1^, and maintained at 305°C for 7.75 min. The injection, transfer line, and ion source temperatures were 280°C, 270°C, and 220°C, respectively. The energy was – 70 eV in the electron impact mode. Mass spectrometric data were acquired in full-scan mode with an *m/z* range of 50–500 at a rate of 20 spectra/s after a solvent delay of 6.1 min.

### DNA extraction, 16S rRNA gene amplification, sequencing, and analysis

Approximately 0.5–1 g of colonic chyme was collected from each sample, and microbial community genomic DNA was extracted from the microbial community according to the manufacturer's instructions for the E.Z.N.A.^®^ soil DNA kit (D5625-02, Omega Bio-Tek, Inc., Norcross, GA, USA). Then, it was stored at – 80°C until the time of analysis. DNA purity and concentration were checked by 1% agarose gel electrophoresis and NanoDrop2000 spectrophotometer (Thermo Fisher Scientific, Waltham, MA, USA), respectively. The V3–V4 regions of the bacterial 16S rRNA gene were amplified with the following primer set: 338F (5′-ACTCCTACGGGAGGCAGCAG-3′) and 806R (5′-GGACTACHVGGGTWTCTAAT-3′). The reaction system included 4 μl of 5 × FastPfu Buffer, 2 μl of 2.5 mM dNTPs, 0.8 μl of each primer (5 μM), 0.4 μl of FastPfu Polymerase, and 10 μl of DNA template. The reactions were performed based on GeneAmp^®^ 9700 (Applied Biosystems, Foster City, CA, USA). The processes were briefly as follows: the denaturation lasted for 3 min at 95°C followed by 27 cycles of 95°C for 30 s; 55°C for 30 s; and 72°C for 45 s, with a final extension of 10 min at 72°C. Meanwhile, the amplified fragments were determined by electrophoresis on a 2% agarose gel. Then, the products were purified with the AxyPrep DNA Gel Extraction kit (Axygen Bioscience, CA, USA) according to the manufacturer's instructions. The raw microbial sequence data were analyzed and processed by Majorbio Bio-Pharm Technology Co. Ltd. (Shanghai, China). The sequences were analyzed and assigned to operational taxonomic units (OTUs; 97% identity). α-diversity whose coverage was based on the Chao 1 within each sample was generated by QIIME (Version 174 1.7.0) (Qingsen, 2017), and β-diversity was estimated by computing the unweighted Unifrac distance and visualized using principal coordinate analysis (PCA).

### Statistical analysis

For multivariate statistical analysis of serum metabolites, normalized data were transformed using the SIMCA 14.1 software package (V14.1, MKS Data Analytics Solutions; Umea, Sweden). Data were subjected to analysis of variance (ANOVA) using SPSS Version 23.0 (IBM Corp; Armonk, New York). Student's *t*-test was used to analyze the differences between the two systems, and the results were presented as means ± standard error of the mean (SEM). The relationships among serum metabolites and bacterial species were explored using Pearson's correlation analysis and a correlation matrix was generated. The results were extracted using GraphPad Prism software 8.0.0 (San Diego, CA, USA). A *p* < 0.05 was considered statistically significant. ^*^Indicates a statistically significant difference (*p* < 0.05); ^**^indicates a highly significant difference (*p* < 0.01); and ^***^indicates a highly significant difference (*p* < 0.001).

## Results

### The estrus rate and number of calves of yaks

**Figure 1:**
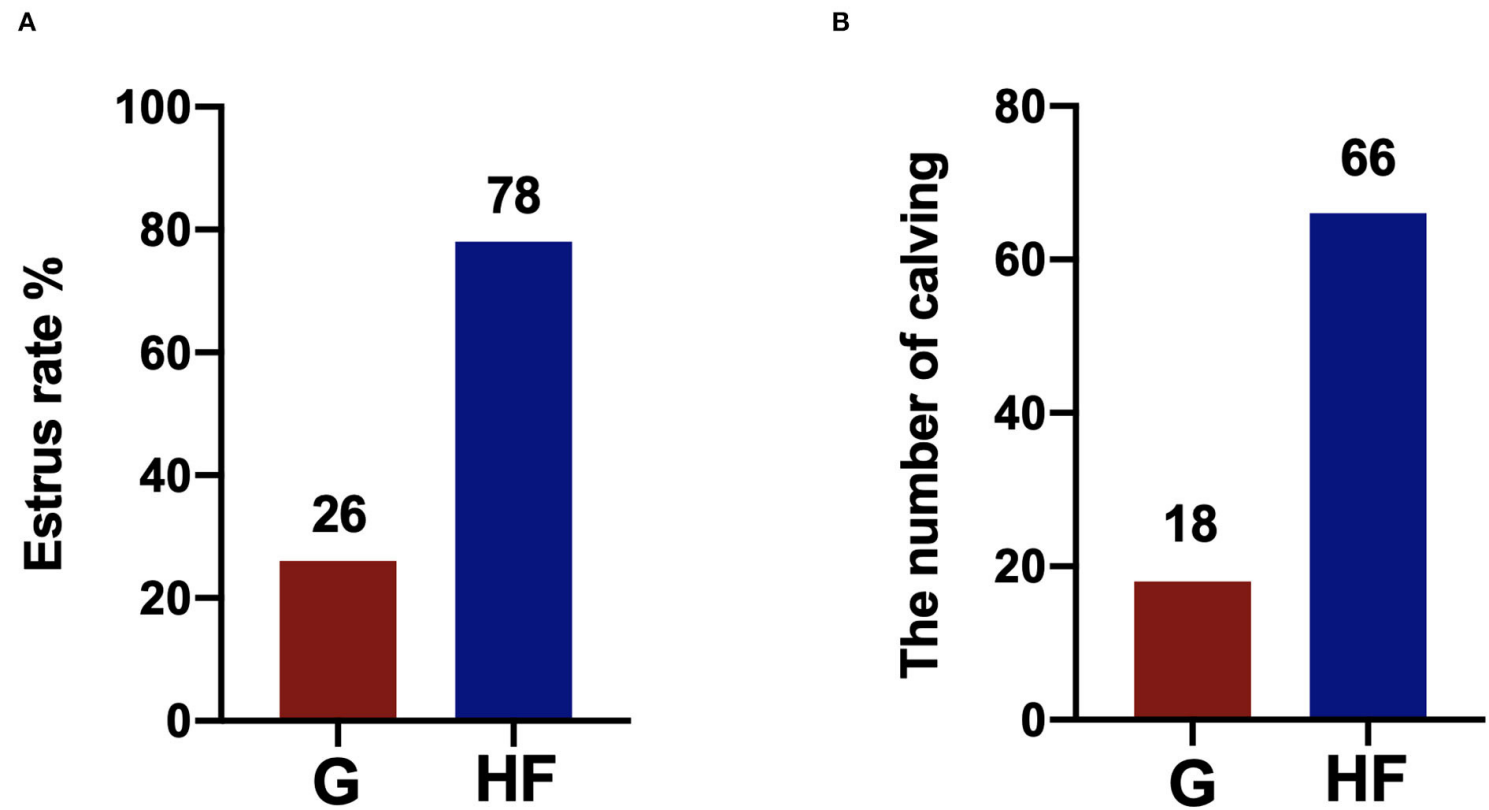
The estrus rate in yaks under the grazing (G) and HF systems. G, grazing system; HF, house feeding system (*n* = 100). **(A)** The estrus rate of yaks. **(B)** The number of calving of yaks.

The estrus rate in yaks is shown in Figures 1A,B. The HF system had a higher estrus rate than the G system. The estrus rate reached 78% in the HF system, which was 52% higher than that in the G system. In addition, the number of calvings was also higher in the HF system than in the G system.

### Metabolomic analysis

**Figure 2:**
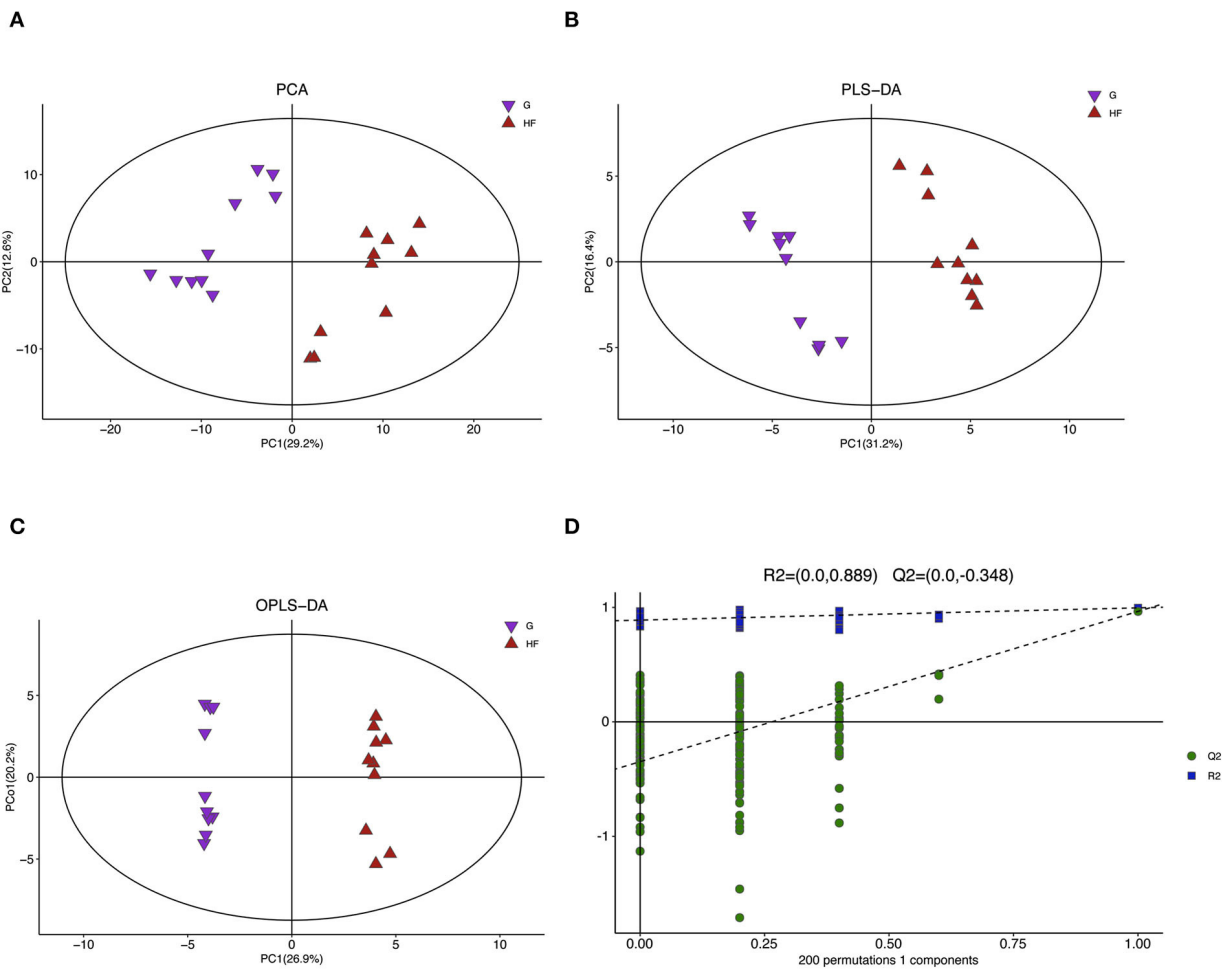
Multivariate analysis model and its cross-validation. G, grazing system; HF, house feeding system. **(A)** Principal component analysis (PCA). **(B)** Partial least squares discrimination analysis (PLS-DA). **(C)** Orthogonal PLS-DA (OPLS-DA). **(D)** Response permutation testing of the model predicted by OPLS-DA. R2X (cum): Cumulative interpretation rate in the x-direction, R2Y (cum): Cumulative interpretation rate in the y-direction, Q2 (cum): Cumulative forecast rate of model, R2 and Q2: Parameters of the response sequencing test, used to measure whether the model is overfitted (*n* = 10).

A total of 775 valid peaks in the serum were identified. Approximately 303 peaks were retained after filtering and de-noising. Most peaks were identified and attributed to endogenous metabolites (similarity > 0) (Supplementary Table S1). To reduce the complexity of the data sets, PCA was applied. The PCA score plot indicated a clear difference in all serum metabolites between the G and HF systems (Figure 2A). The validation plot for the partial least square discrimination analysis (PLS-DA) model revealed that the serum permutation tests were valid (Figure 2B). All samples between the G and HF systems were within the 95% Hotelling's T2 ellipse based on the score plots of the orthogonal PLS-DA (OPLS-DA) model (Figure 2C). Cross-validation with 200 permutation tests indicated that this OPLS-DA model was reliable, with intercepts of R2 and Q2 equal to 0.889 and – 0.384, respectively (Figure 2D).

**Figure 3:**
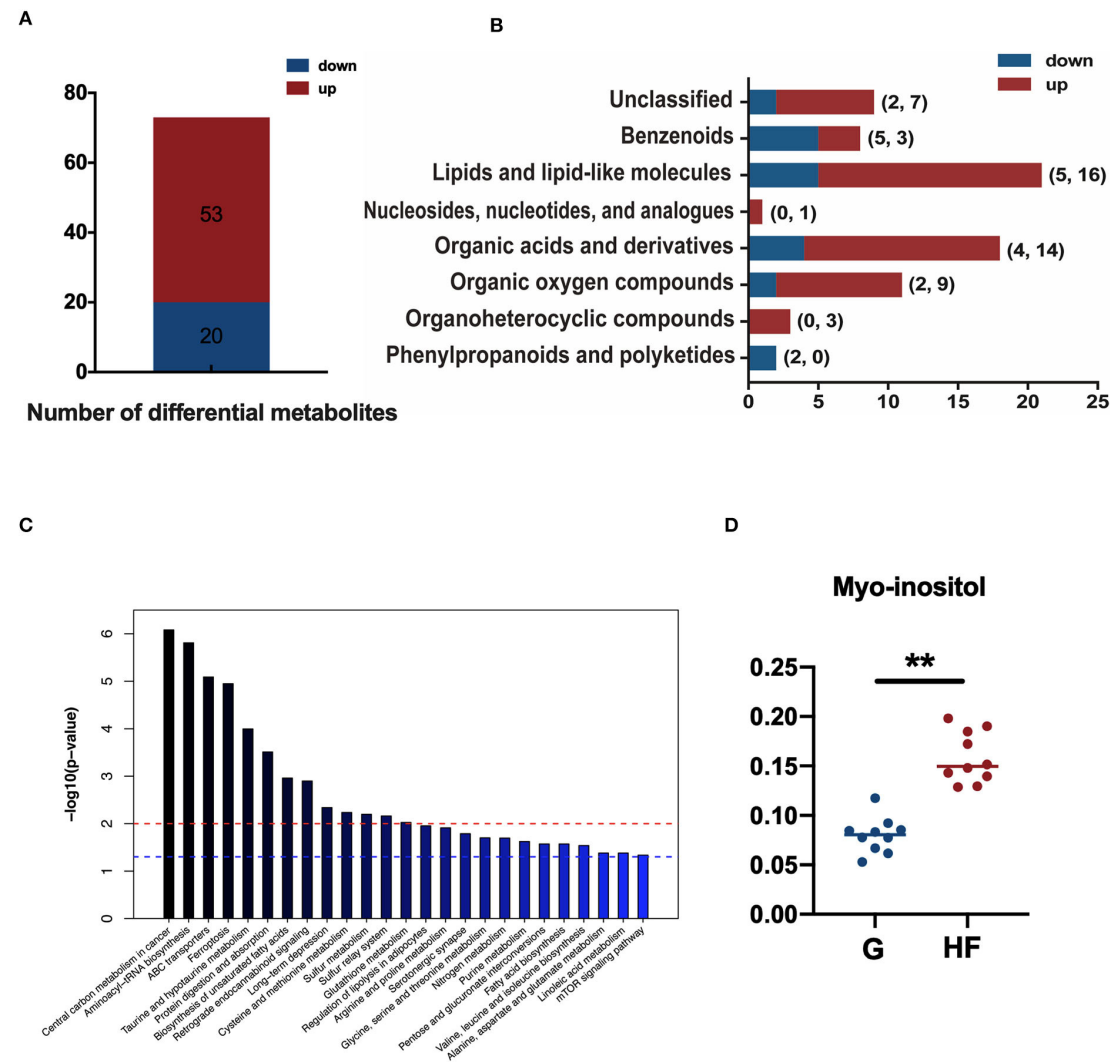
Differentially accumulating metabolites, classification of differential metabolites, and enrichment of pathways between the G and HF systems. G, grazing system; HF, house feeding system. **(A)** The number of differential metabolites between the G and HF systems. **(B)** Classification of differential metabolites. **(C)** Top 25 enriched pathways, red line dotted line shows that *p*-value is 0.01 and blue dotted line shows that *p*-value is 0.05. **(D)** The relative content of myo-inositol in yak serum. Data are expressed as mean ± standard error of the mean (SEM) (*n* = 10).

A total of 73 metabolites were significantly different (*p* < 0.05 and VIP > 1) between the G and HF systems in the total 303 metabolites quantified (Supplementary Table S2). Of these differential metabolites, 53 metabolites were upregulated and 20 metabolites were downregulated in the HF system than in the G system (Figure 3A). Most of the differential metabolites were phenylpropanoids and polyketides, organoheterocyclic compounds, organic oxygen compounds, organic acids and derivatives, nucleosides, nucleotides, and analogs, lipids and lipid-like molecules, benzenoids, and nine other unclassified chemicals (Figure 3B). These results showed that HF notably changed the serum metabolic profile of yaks. A total of 38 differential metabolites were mapped with the Kyoto Encyclopedia of Genes and Genomes (KEGG) database into KEGG pathways (Supplementary Table S3). The KEGG pathway mapper indicated that 25 metabolic signaling pathways were significantly different between the two systems (*p* < 0.05). The top 10 enriched pathways included central carbon metabolism in cancer, aminoacyl–tRNA biosynthesis, and ABC transporters—ferroptosis, taurine, and hypotaurine metabolism, protein digestion and absorption, biosynthesis of unsaturated fatty acids, retrograde endocannabinoid signaling, long-term depression, and the metabolism of cysteine and methionine (Figure 3C). In addition, the relative myo-inositol content was significantly higher in the HF system than in the G system (*p* < 0.01) (Figure 3D).

### Composition of fecal microbiota

**Figure 4:**
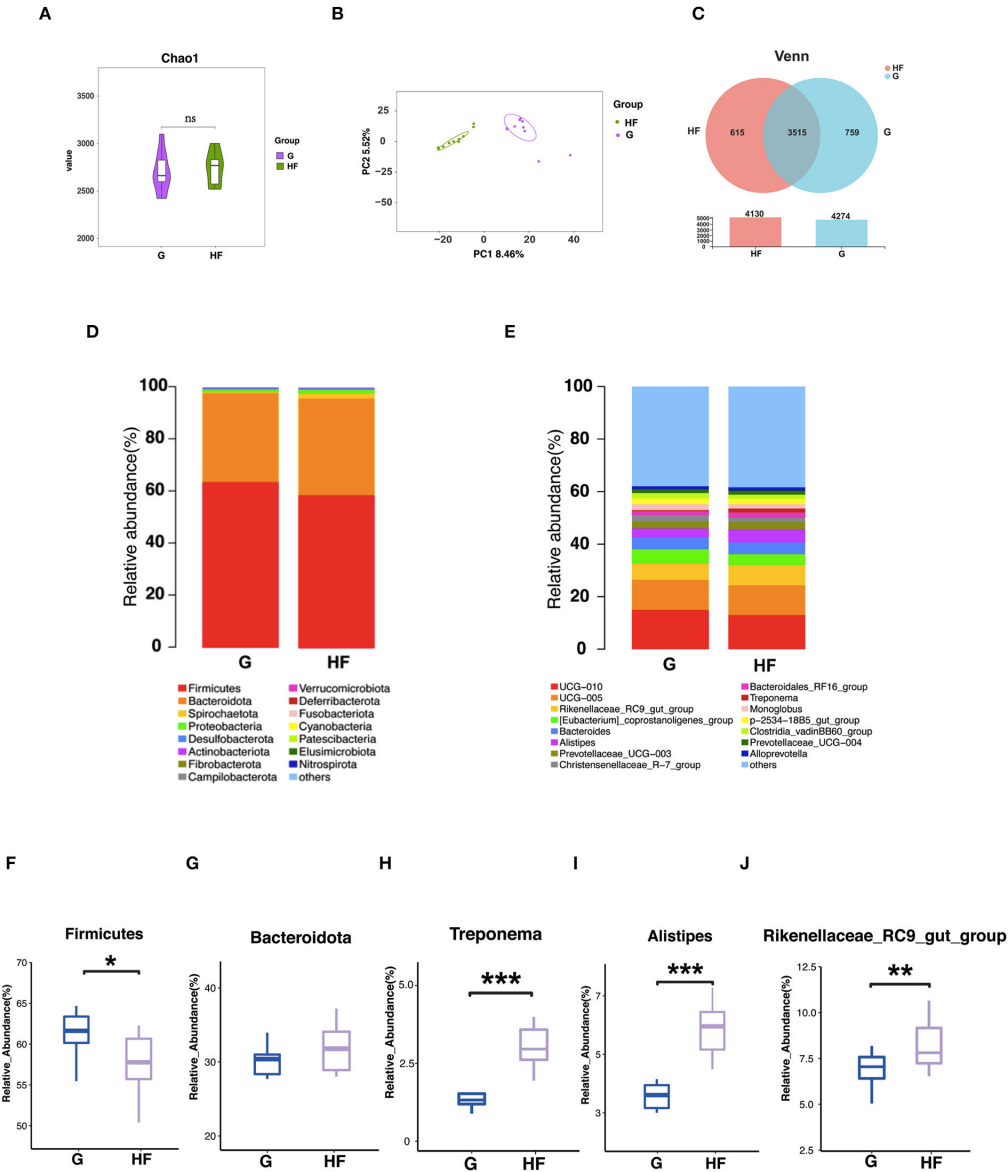
Effects of the grazing and house feeding system on fecal microbiota community composition of yaks. G, grazing system; HF, house feeding system. **(A)** The Chao 1 index of fecal microbiota. **(B)** PC analysis of fecal microbiota at the OUT level. **(C)** The Wayne figures of fecal microbiota. **(D)** Relative abundances of fecal microbiota at the phylum level. **(E)** Relative abundances of fecal microbiota at the genus level. **(F)** Relative abundances of *Firmicutes*. **(G)** Relative abundances of *Bacteroidota*. **(H)** Relative abundances of *Treponema*. **(I)** Relative abundances of *Alistipes*. **(J)** Relative abundances of *Rikenellaceae_RC9_gut_group*. Data are expressed as mean ± SEM (*n* = 10). ^*^Indicates a statistically significant difference (*p* < 0.05), ^**^indicates a highly significant difference (*p* < 0.01), and ^***^indicates a highly significant difference (*p* < 0.001). ns means no significance.

To further assess whether differences in fecal microbiota are the causal factor for differences in serum metabolites between the HF and G systems. Fresh feces were obtained from the HF and G systems, and 16s rRNA gene sequencing analysis was performed. This result showed that there was no significant difference in Chao 1 index between feces from the HF and G systems (Figure 4A). PCA indicated that the HF and G systems were distinctly clustered separately in the distribution of microbiota in feces (Figure 4B). About 615 and 759 OTUs were obtained from the HF and G systems, respectively. A total of 3,515 were common OTUs between the two experimental systems (Figure 4C). Microbial community composition at the phylum and genus level of the two systems is presented in Figures 4E,F. The results showed that fecal samples comprised two major phyla including *Firmicutes* and *Bacteroides* (Figure 4D). At the genus level, the top five most abundant genera in the two systems were *UCG*−*010, UCG*−*005, Rikenellaceae_RC9_gut_group, [Eubacterium]_coprostanoligenes_group*, and *Bacteroides* (Figure 4E). *Firmicutes* were relatively more abundant in the G group than in the HF system (*p* < 0.05) (Figure 4F). The relative abundance of Bacteroidota was also higher in the HF system but not significant (Figure 4G). *Treponema, Alistipes*, and *Rikenellaceae_RC9_gut_group* were relatively more abundant in the HF system than in the G system (*p* < 0.01) (Figures 4H–J).

### Correlation between microbial communities and metabolites of serum

**Figure 5:**
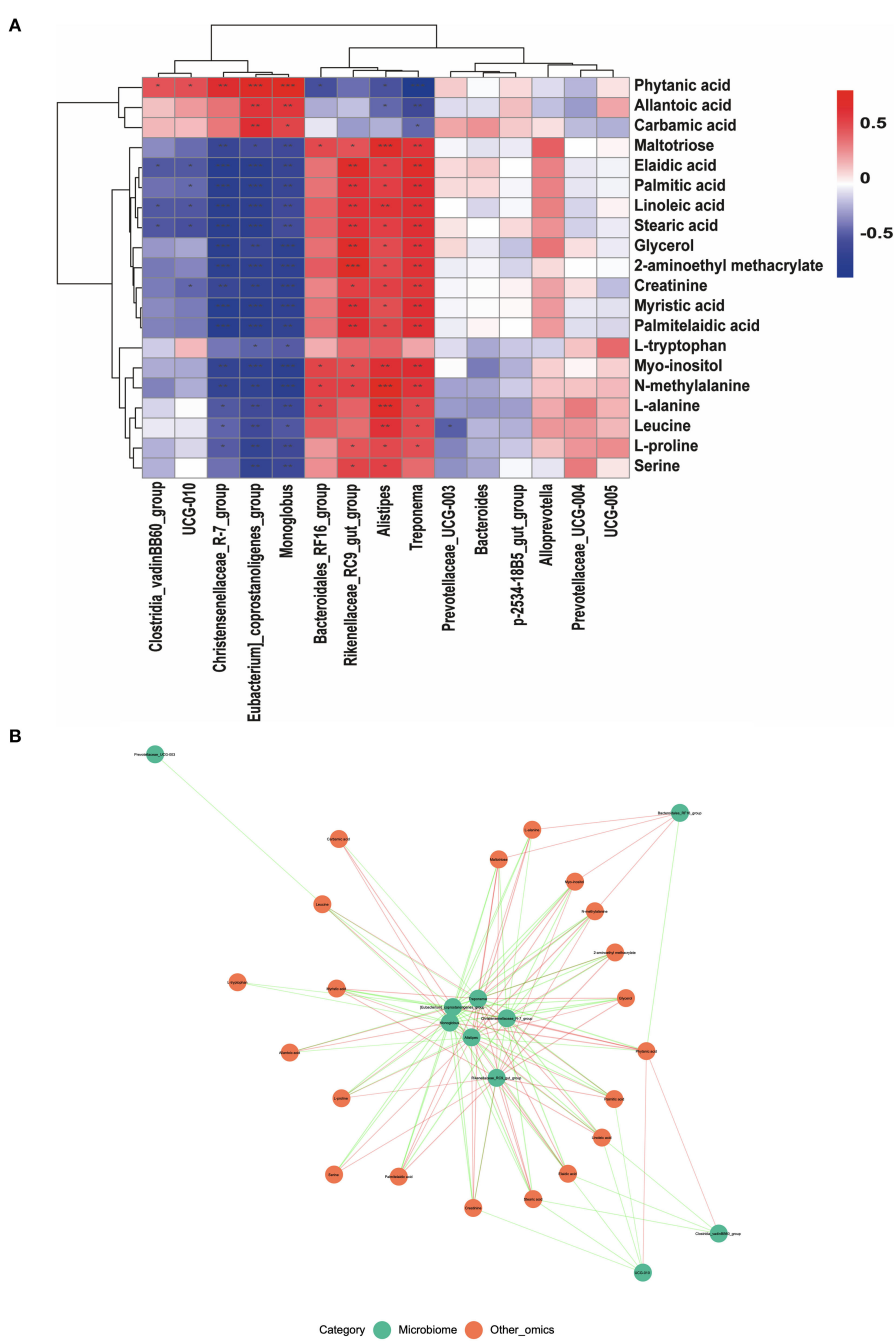
The correlation of fecal microbiota and serum metabolites. **(A)** The pearson's correlation analysis between differential serum metabolites and the top 15 genera. **(B)** The core network figure between differential serum metabolites and the top 10 genera. ^*^Indicates a statistically significant difference (*p* < 0.05), ^**^indicates a highly significant difference (*p* < 0.01), and ^***^indicates a highly significant difference (*p* < 0.001). ns means no significance.

Correlations between differential metabolites and the top 15 genera between the G and HF systems were obtained by Pearson's correlation analysis. As shown in Figure 5, the results showed that, except for phytanic acid, allantoic acid, and carbamic acid, the metabolites were negatively associated with the relative abundance of *Clostridia_vadinBB60_group, UCG-010, Christensenellaceae_R-7_group, [Eubacterium]_coprostanoligenes_group, and Monoglobus* (Figure 5A). Except for phytanic acid, allantoic acid, and carbamic acid, the metabolites were positively associated with the relative abundance of *Bacteroidales_RF16_group, Rikenellaceae_RC9_gut_group, Alistipes, and Treponema* (Figure 5A). The core network figure indicated that metabolites and microbes at the genus level have a significant correlation (*p* < 0.05) (Figure 5B). *Treponema, [Eubacterium]_coprostanoligenes_group, Christensenellaceae_R*−*7_group, Monoglobus, Alistipes*, and *Rikenellaceae_RC9_gut_group* were the core bacteria (Figure 5B).

## Discussion

Yaks are the only bovine species that has adapted well to the Qinghai-Tibetan plateau (Hu et al., 2021). There are approximately 14 million yaks in the Qinghai-Tibetan plateau area of China, accounting for about 90% of the world's yak population (Liang et al., 2021). Yaks are also the main source of income for Tibetan nomadic people (Zou et al., 2020). However, yaks widely exhibit a low estrus rate (Fu et al., 2014b). This severely restricts the economic income of Tibetan herders. In this study, this issue has been well studied, and it was found that the estrus rate in yaks was improved by HF. GC-MS was used to identify differential metabolites in the serum of rutting yaks under different feeding management systems in the present study. Sequencing of the 16S rRNA gene was used to analyze the microbiota in the feces of yaks.

Currently, GC-MS, as a mature technology, has a lot of advantages, which can be used to analyze metabolism products, including organic acids, amino acids, carbohydrates, and fatty acids (Ming et al., 2018). GC-MS has been widely used in many biological fields, including aquaculture (Mabuchi et al., 2018), medicine (Mazumder et al., 2019), food domain (Yang et al., 2018), and livestock (Song et al., 2019). In this study, we found that PCA, PLS-DA, and OPLS-DA clearly separate the G and HF systems from each other. There were significant differences in 73 metabolites (*p* < 0.05 and VIP > 1) between the G and HF systems. Among these metabolites, 53 metabolites were upregulated and 20 metabolites were downregulated in the HF system compared with the G system, and most of them were related to lipids and lipid-like molecules, organic acids and derivatives, and organic oxygen compounds. The KEGG pathway analysis indicated that these metabolites were mainly related to central carbon metabolism in cancer, aminoacyl–tRNA biosynthesis, and ABC transporters. Previous studies found that the majority of metabolites in dairy cows showed differential regulation between the G and HF systems. For example, Ashokan et al. (2021) reported that G and non-grazing systems immensely affected the metabolism of cysteine and selenoamino acids in milk. And, the metabolic pathways of cysteine, methionine, and selenoamino acids were found to be upregulated in non-grazing systems. Meanwhile, Sun et al. (2015) found that two different diets (corn stover and alfalfa hay) had an immense effect on the metabolism of glycine, serine, and threonine in milk. It was previously established that the abovementioned differences were mainly affected by feeding systems (Ashokan et al., 2021). However, differences in feeding systems were mainly embodied with an apparent difference in the ingredient and nutrient levels in diets (Butler et al., 2006). In this study, yaks in the HF system were raised on the formulated diet in a farm; however, yaks in the G system were still in the semi-wild state raised by the natural G system. Hence, the observation of a large difference in the present study might be due to different diets.

The estrous cycle is a cyclical pattern of ovarian activity, which is essential for reproductive health (Cora et al., 2015). Numerous studies have proven that, under estrous condition, there is a dramatic change in body hormones. These hormones include estradiol, progesterone, follicle-stimulating hormone, and luteinizing hormone (Marcondes et al., 2002). Additionally, there are pronounced changes in metabolites and metabolic pathways of blood (Zhao et al., 2020b). For example, Zhao et al. (2020b) found that plasma differential metabolites of inactive ovaries from dairy cows, which were mainly associated with the metabolism of amino acids and carbohydrates, might affect follicular growth through pathway analysis. This result indicated that follicular growth in dairy cows is related to the metabolism of carbohydrates and amino acids using single or multiple pathway(s). In this study, we found that serum metabolites of yaks have also changed tremendously, and the estrus rate reached 78% in the HF system and it was higher than that of G system (52%). Previous studies found that nutritional level has an immense impact on cow estrus as there is a close relationship between ovarian activity and energy balance (Rodrigues et al., 2018). However, up to now, there are no studies on how nutritional level affects serum metabolites when animals were in estrus. Thus, for the time being, we only speculated that this outcome could have been caused by a change in serum metabolites as a result of the difference in nutritional level, which in turn affects the animal's estrus. It is worth noting that in this study we found significantly higher myo-inositol content in the HF system than in the G system. Myo-inositol was found to be involved in the KEGG pathways of ABC transporters. Myo-inositol is polyhydric alcohol with a chemical structure similar to glucose (Showell et al., 2018). It plays an important biological role in mediating the different actions of insulin and is also known as an insulin-sensitizing agent (Showell et al., 2018). Myo-inositol can be considered a dietary supplement and is widely found in beans, fruits, and nuts (Unfer and Porcaro, 2014). Currently, myo-inositol has been proposed as the treatment for ovulatory disorders such as polycystic ovarian syndrome. For example, Kamenov et al. (2015) showed that myo-inositol was well tolerated and may be effective in inducing ovulation in women. Simi et al. (2017) found that myo-inositol can improve ovarian function, oocyte quality, and embryo and pregnancy rates. In addition, Zhao et al. (2020a) also found that the content of myo-inositol was higher in the estrus cow serum than in cows with inactive ovaries. This is similar to the results obtained in this study. Therefore, this result obtained for higher estrus rate in the HF system was also explained using higher myo-inositol content in the HF system.

Intestinal microbiota is essential to maintain health (Bolte et al., 2021). A number of studies have discovered that in ruminant research, the fecal microbiome of cattle plays a critical role not only in animal health and nutrient utilization, but also in production (Shanks et al., 2011). Previous studies found that intestinal microbial composition is influenced by many factors, including genotypes, altitudes, anddiets – especially diets (Li and Zhao, 2015; Mejía-León and Barca, 2015; Lan et al., 2017). In this study, we found that the G system results in an increase in the relative abundance of *Firmicutes*. And, microbiota diversity differed between the G and HF systems. These differences were also reported in previous studies (Ashokan et al., 2021). This may result from the fact that yaks in the HF system were raised by the formulated diet on a farm and yaks in the G system were still in the semi-wild state raised by the natural G system. There is a positive correlation of relative abundance of *Firmicutes* intake with fiber intake (Chang et al., 2020). Therefore, there is a probability that the observation of a large difference in the present study may be due to the different diets. Moreover, at the genus level, a large number of microbiota with differential abundance were also identified, including *Rikenellaceae_RC9_gut_group, [Eubacterium]_coprostanoligenes_group, Alistipes, Monoglobus, Treponema*, and *Christensenellaceae_R*−*7_group*. These discrepancies could be also due to the differences in dietary composition (Guo et al., 2019).

As is well-known, the variability of serum metabolites could be greatly influenced by the gut microbiota (Guertin et al., 2014). Therefore, to obtain more comprehensive information on the effect of the G and HF systems on the estrus rate in yaks. Associations between serum metabolites and specific gut microbiota in fresh fecal samples were assessed by Spearman's rank correlation analysis. We found that *Treponema, [Eubacterium]_coprostanoligenes_group, Christensenellaceae_R*−*7_group, Monoglobus, Alistipes, and Rikenellaceae_RC9_gut_group* become the core bacteria as they are particularly relevant to serum metabolites. Studies were well reported that *Treponema, Alistipes*, and *Rikenellaceae_RC9_gut_group* are acetate producers (Stanton, 1984; Oliphant and Allen-Vercoe, 2019; Sha et al., 2021), and have revealed the relationship between acetate and myo-inositol transformation-associated (Bui et al., 2021). Therefore, this also revealed that an increase in the estrus rate in the HF system may be partly attributed to the specific fecal microbiota producing more acetates, which in turn promotes myo-inositol products.

**Figure 6:**
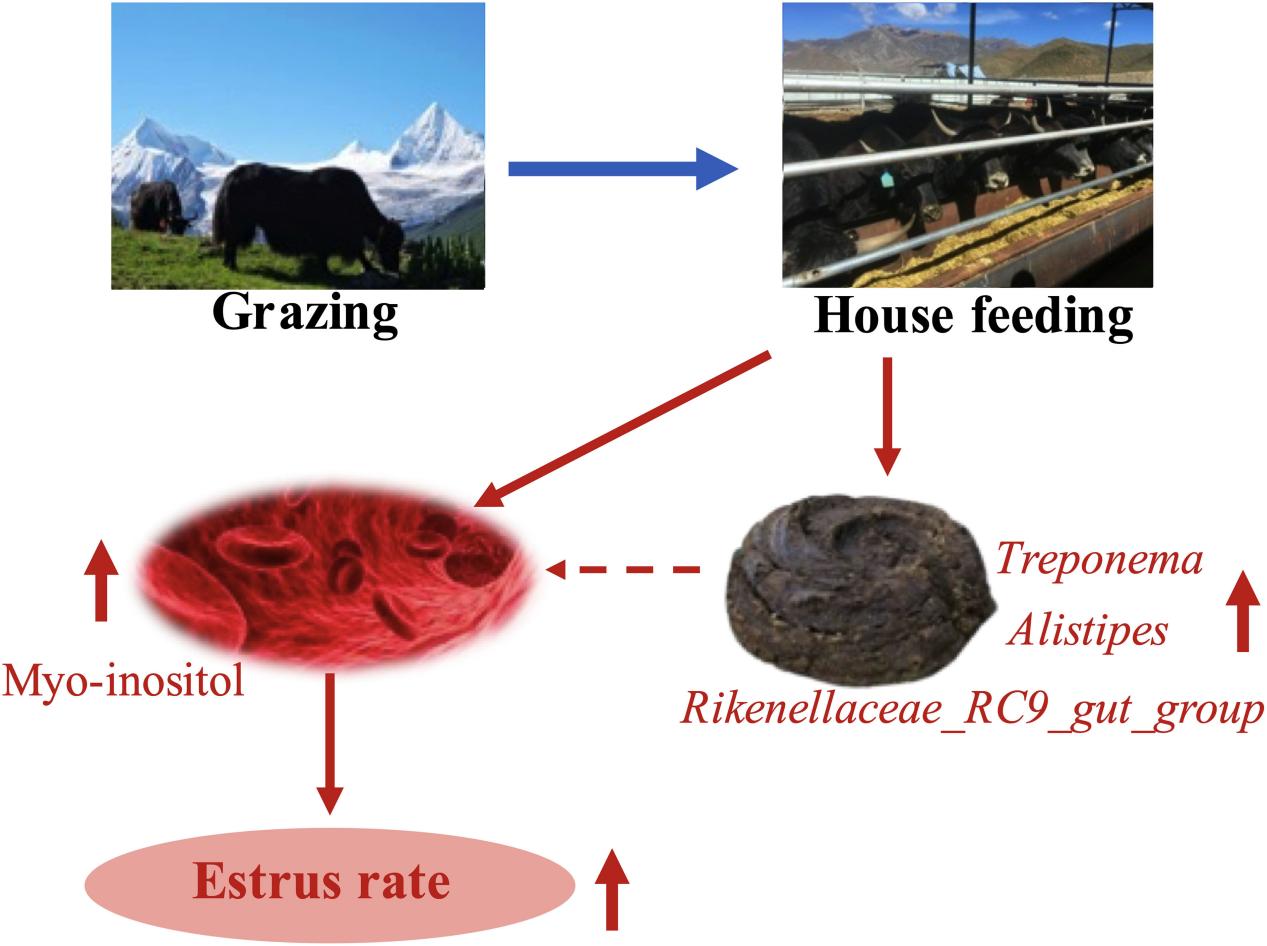
HF system improves the estrus rate in yaks by increasing specific gut microbes and myo-inositol content in serum.

In summary, the HF mode promoted the estrus rate and changed the composition of fecal microbiota and serum metabolites in yaks. Increased estrus rate may be obtained due to enhanced myo-inositol content in the serum of yaks *via* the HF mode. Correlation analysis suggested that myo-inositol content might be partly increased *via* specific fecal microbiota in yaks, contributing to the estrus rate (Figure 6). Unfortunately, we were unable to collect yak ovary samples. The findings of the present study may provide a novel therapeutic strategy for low estrus rate in G yaks. To further elucidate this mechanism, we would investigate further in this direction.

## Data availability statement

The datasets presented in this study were submitted to the NCBI Sequence Read Archive (SRA) database. The name of the repository and accession number is [PRJNA871084].

## Ethics statement

Ethical review and approval was not required for the animal study because This research carries no risk of harm to animals and will not affect their rights or welfare.

## Author contributions

YZ and XL designed this experiment. Lz, Sz, Suolang, and Ciyang carried out this experiment. YZ, GS, and Cy wrote this manuscript. Bw revised this manuscript. All authors reviewed this manuscript.

## Funding

This research was supported by the Breeding and Efficient Propagation of Yaks in Gesangtang of Linzhou County (QYXTZX-LS2020-01), Seed Industry Innovation and Healthy Breeding of Yaks (XZ202101ZD0002N), and National Meat Yaks Industry Technology System (CARS-37).

## Conflict of interest

The authors declare that the research was conducted in the absence of any commercial or financial relationships that could be construed as a potential conflict of interest.

## Publisher's note

All claims expressed in this article are solely those of the authors and do not necessarily represent those of their affiliated organizations, or those of the publisher, the editors and the reviewers. Any product that may be evaluated in this article, or claim that may be made by its manufacturer, is not guaranteed or endorsed by the publisher.
